# Supplementary material for: Identification of Freezing-Responsive microRNAs and Their Targets in Chinese Jujube by Small RNA and Degradome Sequencing
Source: Int J Mol Sci. 2026 Sep 4;27(17):7912. doi: 10.3390/ijms27177912 (PMC13566890; doi:10.3390/ijms27177912)
Supplement: Supplementary file 1 [file ijms-27-07912-s001.zip › Supplementary Material S2/novel_miR_2.pdf]

Provisional ID : novel_miR_2
Score total : 0.5
Score for star read(s) : -1.3
Score for read counts : 0
Score for mfe : 0.2
Score for randfold : 1.6
Score for cons. seed :
Total read count : 360
Mature read count : 360
Loop read count : 0
Star read count : 0

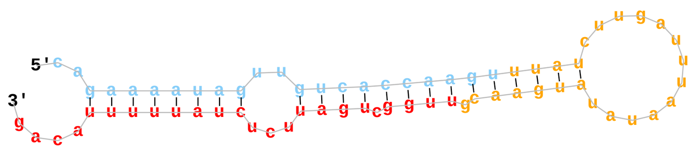

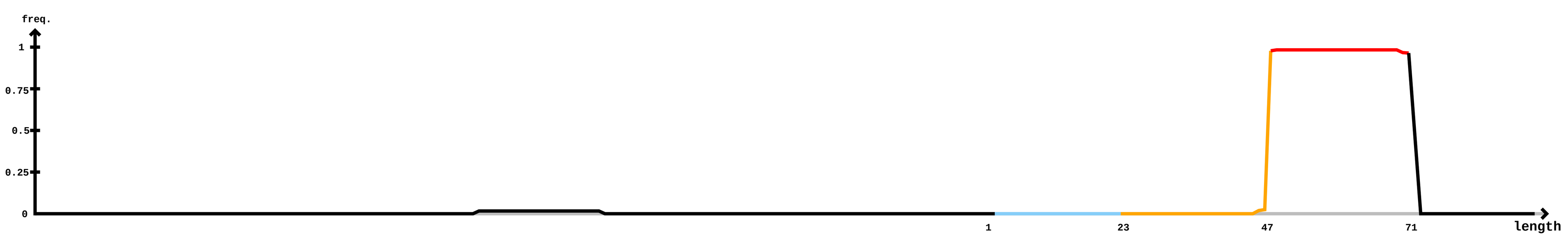

Star Mature

```
5'- cuugacaaguuacauuacgugaaugaauuauuuguuuaugguaaugaguuuucagauuaguuagucaucuuacaacucuuuaaauucguuaugauugcuaaaaaugucaaugccaugucuacuuugauuuguuaaaaguuccauucaguaaggccuuucagaaaauaguugucaccaaguuuaucuugauuuaauauaugaacguuggcugauucucuauuuuuacaggcaaugaauggucaauaucuuu -3'   exp
    .((((((((((....(((((..(((....((((...((.((((((((((..(((...((.((....)).))...)))..)))))))))))).))...))))....)))....)))))......)))))))))).....(((((((.....((((....((((((((..(((((((((((((............)))))).)))).))))...))))))))..))))..)))))))..........   reads   mm   sample
    ........................................................................caacucuuuaaauucguuaug.............................................................................................................................................................   6   0   LCK-3
    ..........................................................................................................................................................................................................cguuggcugauucucuauuuuuacag......................   3   0   LT-1
    ..........................................................................................................................................................................................................cguuggcugauucucuauuuuuacag......................   1   0   JCK-2
    ..........................................................................................................................................................................................................cguuggcugauucucuCuuuuuacag......................   2   1   JCK-3
    ..........................................................................................................................................................................................................cguuggcugauucucuauuuuuacag......................   1   0   JCK-3
    ...........................................................................................................................................................................................................guuggcugauucucuauuuuuacag......................   2   0   LT-3
    ............................................................................................................................................................................................................uuggcugauucucuauuuuuac........................   3   0   JCK-3
    ............................................................................................................................................................................................................uuggcugauucucuauuuuuac........................   2   0   JCK-2
    ............................................................................................................................................................................................................uuggcugauucucuauuuuuac........................   1   0   LT-3
    ............................................................................................................................................................................................................uuggcugauucucuauuuuuaca.......................   1   0   LT-1
    ............................................................................................................................................................................................................uuggcugauucucuauuuuuacag......................   40   0   JCK-1
    ............................................................................................................................................................................................................uuggcugauucucuCuuuuuacag......................   1   1   LCK-3
    ............................................................................................................................................................................................................uuggcugauucucuauuuuuacag......................   8   0   JT-3
    ............................................................................................................................................................................................................uuggcugauucucuauuuuuacag......................   110   0   LCK-3
    ............................................................................................................................................................................................................uuggcugauucucuauuuuuacag......................   43   0   LT-2
    ............................................................................................................................................................................................................uuggcugauucucuauuuuuacag......................   19   0   LT-3
    ............................................................................................................................................................................................................uuggcugauucucuauuuuuacag......................   3   0   JT-1
    ............................................................................................................................................................................................................uuggcugauucucuauuuuuacag......................   34   0   LT-1
    ............................................................................................................................................................................................................uuggcugauucucuauuuuuacag......................   39   0   JCK-3
    ............................................................................................................................................................................................................uuggcugauucucuauuuuuacag......................   40   0   JCK-2
    ............................................................................................................................................................................................................uuggcugauucucuauuuuuacag......................   4   0   JT-2
    ............................................................................................................................................................................................................uuggcugCuucucuauuuuuacag......................   1   1   LCK-3
    .............................................................................................................................................................................................................uggcugauucucuauuuuuacag......................   1   0   JCK-3
    .............................................................................................................................................................................................................uggcugauucucuauuuuuacag......................   1   0   JCK-1
```
